# Supplementary material for: SpatialArtifacts: a computational framework for tissue artifact detection in spatial transcriptomics data
Source: bioRxiv. 2026 May 18:2026.05.15.725260. Preprint. [Version 1] doi: 10.64898/2026.05.15.725260 (PMC13228377; doi:10.64898/2026.05.15.725260)
Supplement: Supplement 1 [file NIHPP2026.05.15.725260v1-supplement-1.pdf]

bioRxiv preprint doi: https://doi.org/10.64898/2026.05.15.725260; this version posted May 18, 2026. The copyright holder for this preprint (which was not certified by peer review) is the author/funder, who has granted bioRxiv a license to display the preprint in perpetuity. It is made available under a CC-BY 4.0 International license.

Supplementary Materials

SpatialArtifacts: a computational framework for tissue artifact detection in spatial transcriptomics data

Jiali Harriet He, Jacqueline R. Thompson, Michael Totty, Stephanie C. Hicks*

*Correspondence to shicks19@jhu.edu

Contents

1. **Supplementary Table S1**
2. **Supplementary Figures S1-S6**

bioRxiv preprint doi: https://doi.org/10.64898/2026.05.15.725260; this version posted May 18, 2026. The copyright holder for this preprint (which was not certified by peer review) is the author/funder, who has granted bioRxiv a license to display the preprint in perpetuity. It is made available under a CC-BY 4.0 International license.

Dataset	Platform	Method	Total Spots	Artifacts	% Removed
Hippocampus	Standard	BLADE	4,965	1,101	22.18%
		SpotSweeper	4,965	11	0.22%
		**SpatialArtifacts**	4,965	**167**	**3.36%**
DLPFC	Standard	BLADE	3,529	461	13.06%
		SpotSweeper	3,529	33	0.94%
		**SpatialArtifacts**	3,529	**87**	**2.47%**
VisiumHD (16μm)	HD	BLADE	137,051	7,680	5.60%
		SpotSweeper	137,051	813	0.59%
		**SpatialArtifacts**	137,051	**2,632**	**1.92%**

Table S1: Quantitative benchmarking of artifact detection methods across platforms and tissue types

bioRxiv preprint doi: https://doi.org/10.64898/2026.05.15.725260; this version posted May 18, 2026. The copyright holder for this preprint (which was not certified by peer review) is the author/funder, who has granted bioRxiv a license to display the preprint in perpetuity. It is made available under a CC-BY 4.0 International license.

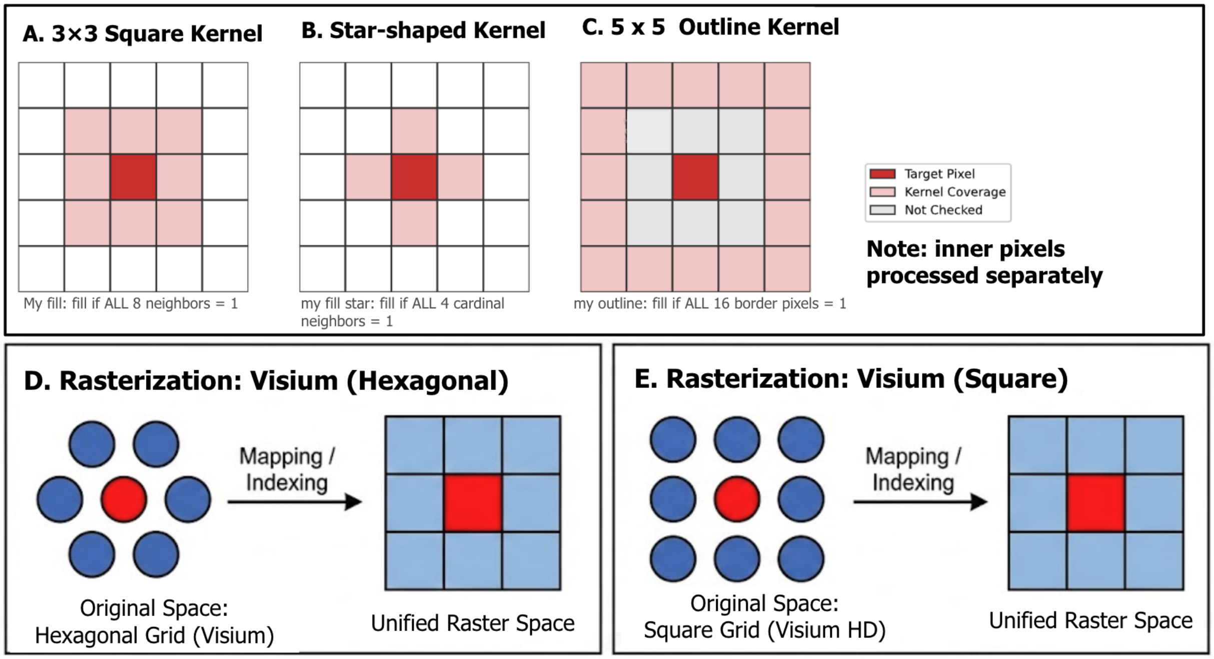

Figure S1: Overview of computational approaches for morphological pattern recognition. (**A-C**) Schematic of the focal filling kernels used for artifact reconstruction, including the 3×3 square kernel, star-shaped cardinal neighbor kernel, and 5×5 outline kernel. (**D-E**) Illustration of the rasterization process used to map hexagonal (standard Visium) and square (VisiumHD) spot coordinates into a unified raster space for morphological processing.

bioRxiv preprint doi: https://doi.org/10.64898/2026.05.15.725260; this version posted May 18, 2026. The copyright holder for this preprint (which was not certified by peer review) is the author/funder, who has granted bioRxiv a license to display the preprint in perpetuity. It is made available under a CC-BY 4.0 International license.

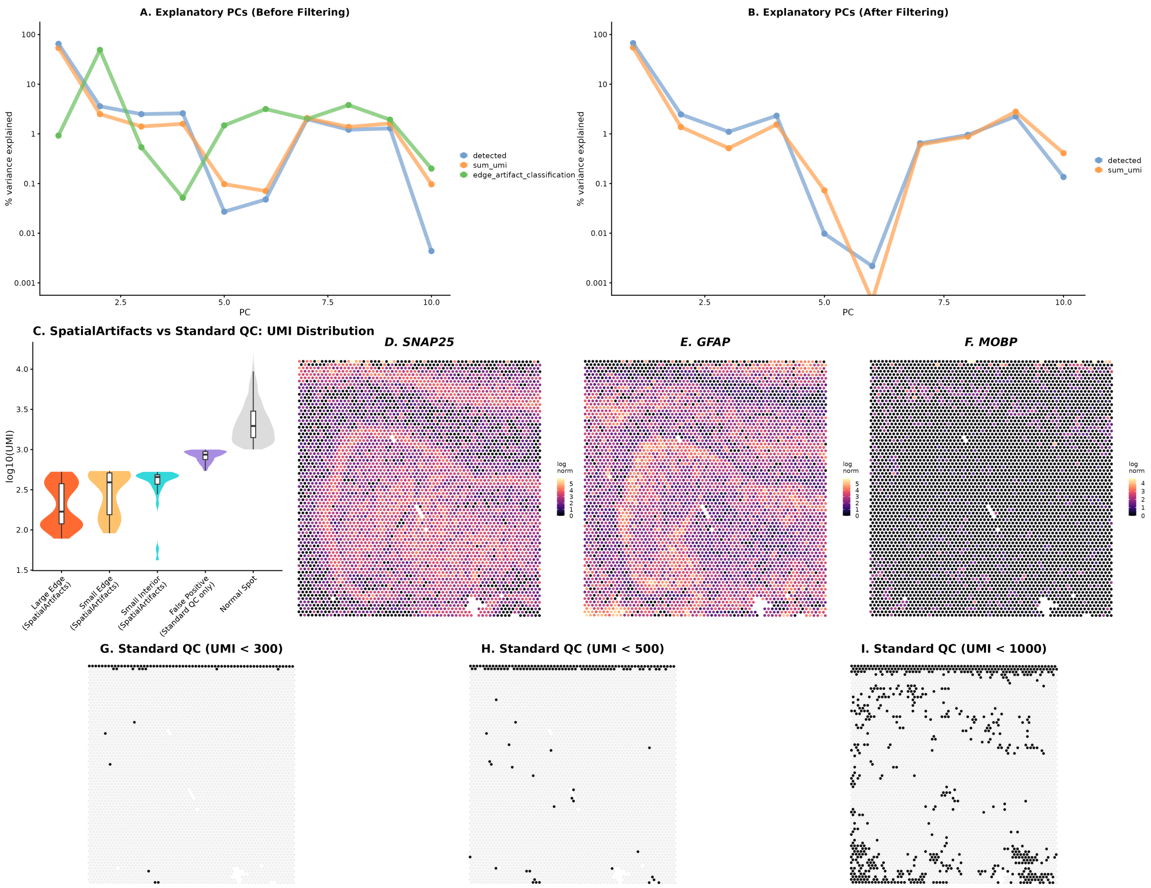

Figure S2: Spot-level PCA, QC validation, and threshold comparison for human hippocampus sample V11L05-335_C1. (**A**–**B**) Assessment of metadata variable contributions to the top 10 principal components using `plotExplanatoryPCs`, before (A) and after (B) artifact removal. Prior to filtering, artifact classification explained a substantial proportion of variance in PC1 and PC2. After removing artifact spots, the contribution of QC metrics to mid-range PCs (PC4–6) decreased by approximately 100-fold, suggesting that these components previously captured artifact-driven technical variation.(**C**) UMI distribution comparison between SpatialArtifacts artifact categories and spots flagged exclusively by standard global thresholding (UMI $<$ 1000). The 492 false positive spots flagged only by standard QC show a median UMI of 840, consistent with biologically meaningful low-expression regions rather than technical artifacts.(**D**–**F**) Spatial expression of hippocampal marker genes SNAP25 (D), GFAP (E), and MOBP (F), confirming preserved biological signal in interior regions with naturally lower UMI counts.(**G**–**I**) Comparison of three fixed QC thresholds (UMI $<$ 300, UMI $<$ 500, and UMI $<$ 1000).

bioRxiv preprint doi: https://doi.org/10.64898/2026.05.15.725260; this version posted May 18, 2026. The copyright holder for this preprint (which was not certified by peer review) is the author/funder, who has granted bioRxiv a license to display the preprint in perpetuity. It is made available under a CC-BY 4.0 International license.

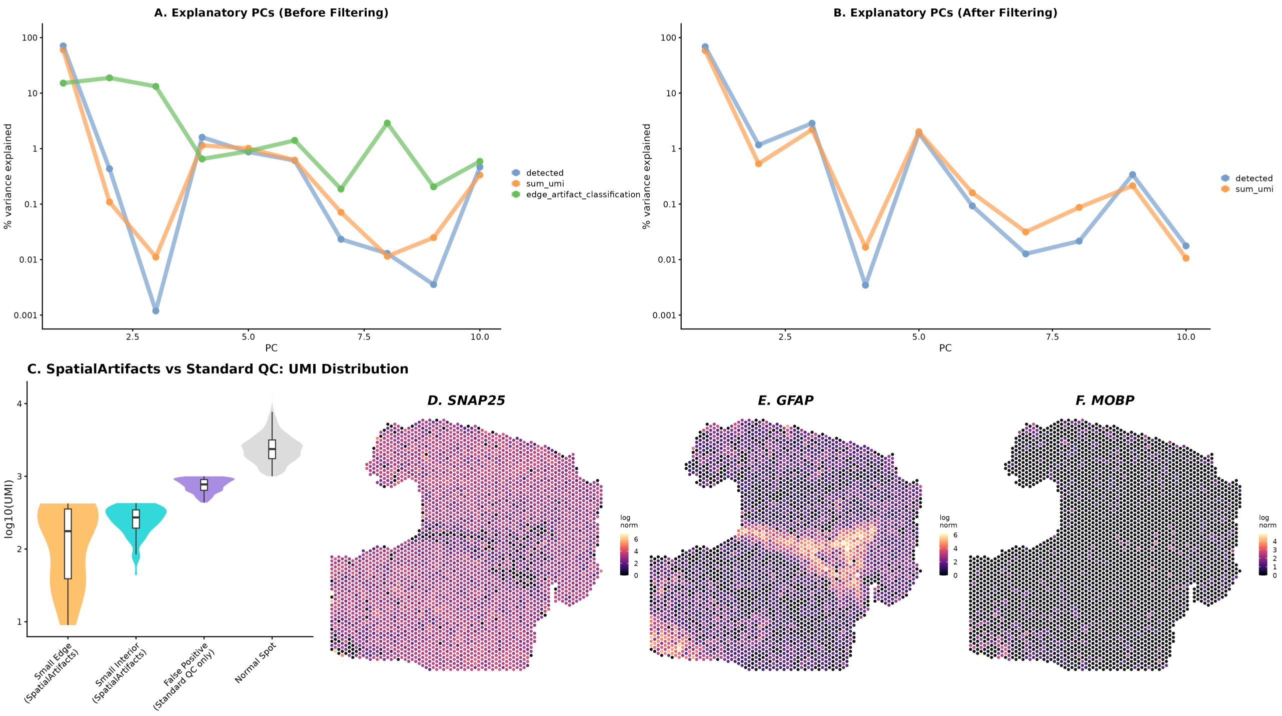

Figure S3: Spot-level PCA and QC validation for DLPFC sample Br8325_ant. (**A–B**) Assessment of metadata variable contributions to the top 10 principal components using `plotExplanatoryPCs`, before (A) and after (B) artifact removal. Prior to filtering, artifact classification explained a substantial proportion of variance in PC1 and PC2. After removal, the contribution of QC metrics was reduced, suggesting that these components previously captured artifact-driven technical variation.(**C**) UMI distribution comparison between SpatialArtifacts artifact categories and spots flagged exclusively by standard global thresholding (UMI $<$ 1000). The 389 false positive spots flagged only by standard QC show a median UMI of 773, consistent with biologically meaningful low-expression regions such as white matter.(**D–F**) Spatial expression of cortical marker genes SNAP25 (D), GFAP (E), and MOBP (F). Artifact spots retained partial SNAP25 expression (median = 2.84) but showed complete loss of GFAP expression (median = 0), consistent with selective technical degradation rather than complete signal loss.

bioRxiv preprint doi: https://doi.org/10.64898/2026.05.15.725260; this version posted May 18, 2026. The copyright holder for this preprint (which was not certified by peer review) is the author/funder, who has granted bioRxiv a license to display the preprint in perpetuity. It is made available under a CC-BY 4.0 International license.

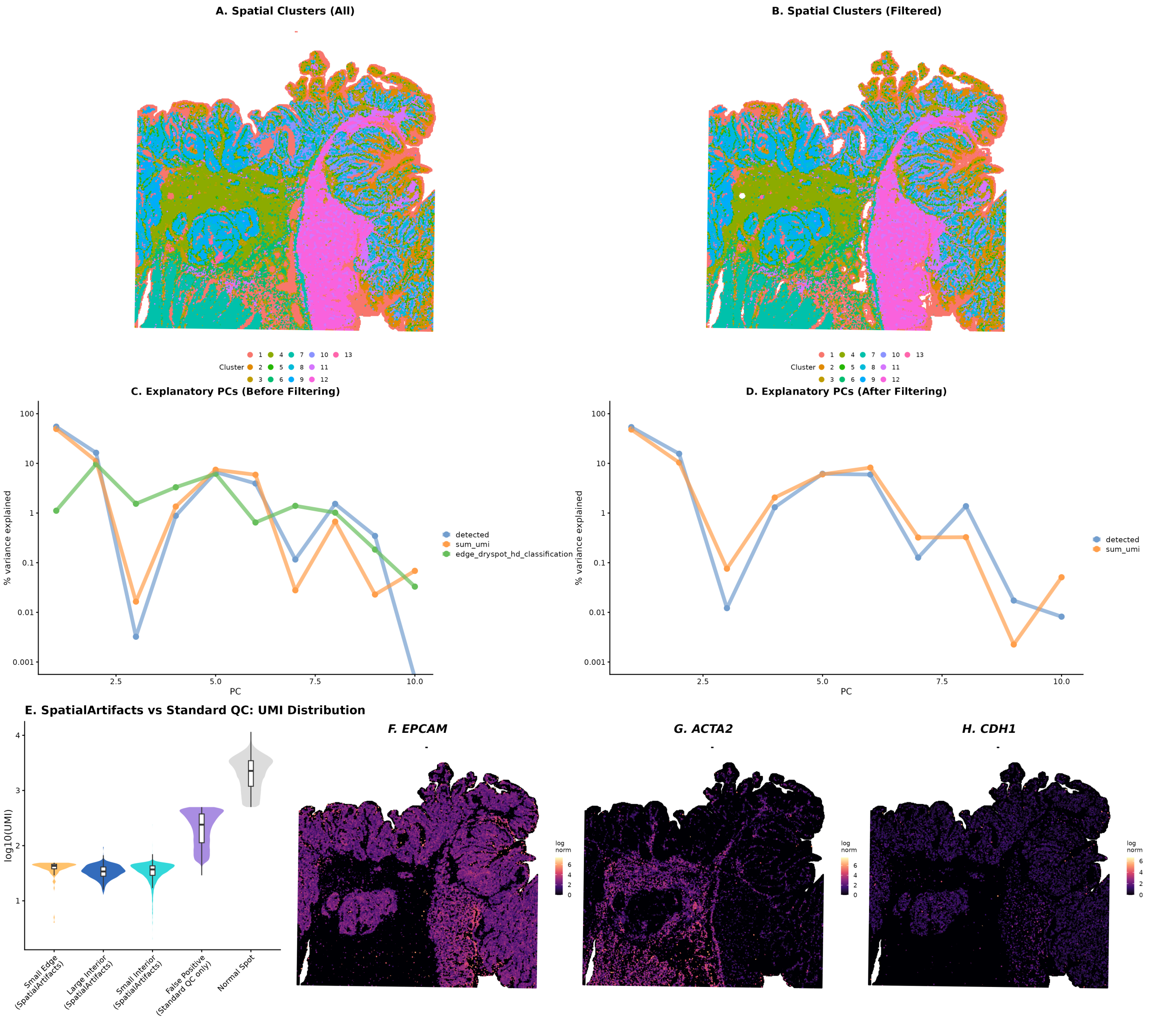

Figure S4: Comprehensive validation on high-resolution VisiumHD colorectal cancer data. (**A–B**) Spatial cluster maps before (A) and after (B) artifact removal, showing improved domain detection with all clusters retained.(**C–D**) Assessment of metadata variable contributions to the top 10 principal components using `plotExplanatoryPCs`, before (C) and after (D) artifact removal. Prior to filtering, artifact classification explained a substantial proportion of variance; after removal, QC metric contributions were reduced, confirming biological specificity of artifact detection. (**E**) UMI distribution comparison between SpatialArtifacts artifact categories and bins flagged exclusively by standard global thresholding (UMI $<$ 500). The 28,186 false positive bins flagged only by standard QC show a median UMI of 239, consistent with biologically meaningful low-expression regions rather than technical artifacts.(**F–H**) Spatial expression of colorectal tissue marker genes EPCAM (F), ACTA2 (G), and CDH1 (H), confirming preserved biological signal in regions with naturally lower UMI counts that are correctly retained by SpatialArtifacts.

bioRxiv preprint doi: https://doi.org/10.64898/2026.05.15.725260; this version posted May 18, 2026. The copyright holder for this preprint (which was not certified by peer review) is the author/funder, who has granted bioRxiv a license to display the preprint in perpetuity. It is made available under a CC-BY 4.0 International license.

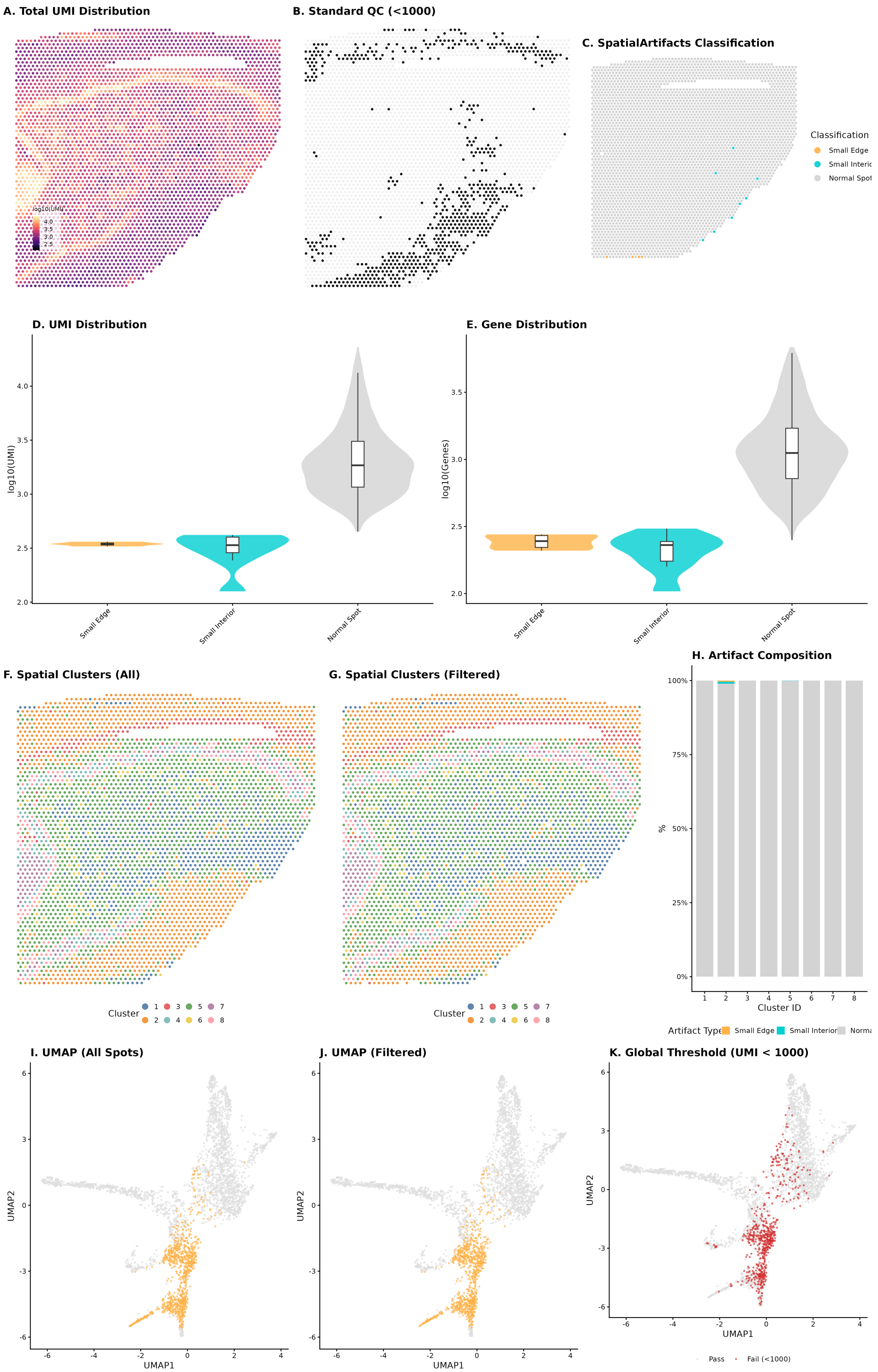

Figure S5: Negative control analysis using a high-quality human hippocampus sample. Evaluation of Sample V11U08-081_B1, which exhibits minimal technical damage. (**A**) Heat map of total UMI ($\log_{10}$ scale). (**B**) Spots flagged as low-quality (black) using standard global QC thresholds (UMI $<$ 1000). (**C**) SpatialArtifacts classification confirming detection of only 12 artifact spots (4 Small Edge and 8 Small Interior). (**D–E**) Violin plots for the distribution of UMI counts (D) and number of detected genes (E). (**F–G**) Spatial clustering before (F) and after (G) artifact removal, demonstrating maintenance of biological structure with all 8 clusters retained. (**H**) Artifact composition analysis revealing less than 1% artifact contamination across all clusters. (**I–J**) UMAPs before (I) and after (J) filtering, confirming no spurious cluster removal. (**K**) Global thresholding comparison (UMI $<$ 1000, red points) removes substantially more spots than SpatialArtifacts.

bioRxiv preprint doi: https://doi.org/10.64898/2026.05.15.725260; this version posted May 18, 2026. The copyright holder for this preprint (which was not certified by peer review) is the author/funder, who has granted bioRxiv a license to display the preprint in perpetuity. It is made available under a CC-BY 4.0 International license.

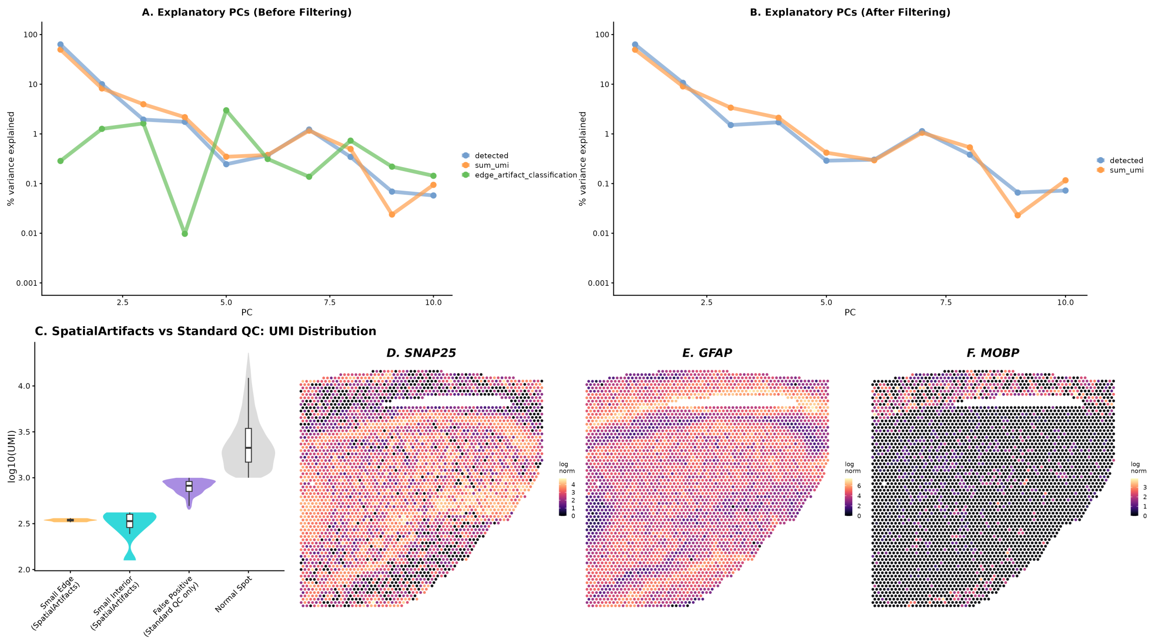

Figure S6: Spot-level PCA and QC validation for negative control Sample V11U08-081_B1. (**A–B**) Assessment of metadata variable contributions to the top 10 principal components using `plotExplanatoryPCs`, before (A) and after (B) artifact removal. The variance structure remains stable, with artifact classification explaining minimal variance, confirming that SpatialArtifacts does not remove biologically meaningful variation in high-quality tissue. (**C**) UMI distribution comparison between SpatialArtifacts artifact categories and spots flagged exclusively by standard global thresholding (UMI $<$ 1000). The 618 false positive spots flagged only by standard QC show a median UMI of 818, consistent with biologically meaningful low-expression regions rather than technical artifacts. (**D–F**) Spatial expression of hippocampal marker genes SNAP25 (D), GFAP (E), and MOBP (F), showing preserved expression across the tissue interior, further confirming the absence of significant technical damage in this sample.
